# Subclinical and latent cardiac dysfunction in obstructive sleep apnea and effectiveness of continuous positive airway pressure

**DOI:** 10.1007/s11325-022-02774-0

**Published:** 2022-12-30

**Authors:** Takahiro Kanda, Kei Tawarahara, Haruta Kato, Humimaro Ishibashi, Naoki Nakamura, Yuki Tokonami, Gaku Matsukura, Mariko Ozeki, Hiroshi Ukigai, Ryosuke Takeuchi

**Affiliations:** Department of Internal Medicine, Division of Cardiology, Hamamatsu Red Cross Hospital, Shizuoka, Japan

**Keywords:** Obstructive sleep apnea, Global longitudinal strain, Continuous positive airway pressure therapy

## Abstract

**Purpose:**

Obstructive sleep apnea (OSA) is associated with various cardiovascular disorders. This study aimed to investigate the effects of OSA on left ventricular (LV) function in patients with OSA who were at risk for heart failure but who had not yet developed structural heart changes. The study also sought to determine the effects of continuous positive airway pressure (CPAP) in these patients.

**Methods:**

In a retrospective study, consecutive patients with polysomnographic OSA (apnea-hypopnea index [AHI] >5) were categorized into mild (AHI < 15), moderate (15 ≤ AHI < 30), and severe OSA (AHI ≥ 30) groups. The subjects were patients with OSA and at risk for heart failure who had not yet developed structural heart changes. All study participants underwent echocardiography and two-dimensional speckle tracking analysis, and their global longitudinal strain (GLS) was calculated.

**Results:**

Of 275 patients, there were 31 with mild, 92 with moderate, and 152 with severe OSA. Of patients with moderate to severe OSA (AHI ≥ 20), 206 started CPAP and 92 patients underwent follow-up echocardiogram and speckle tracking echo analysis (median period of CPAP use: 283 days [258 to 391]). GLS was significantly reduced in patients with moderate and severe OSA compared with mild OSA (−17.8±3.1 vs. −18.0±2.6 vs. −19.3±2.8%, *p*=0.038). The proportion of patients with GLS ≥ −18% was significantly higher among the patients with moderate to severe OSA than among those with mild OSA. GLS improved after CPAP therapy in patients with moderate to severe OSA (GLS: −18.1±2.7% to −19.0±2.8%, *p*=0.004). Significant improvement in GLS was confirmed, particularly among patients with good CPAP adherence.

**Conclusion:**

Moderate to severe OSA is associated with LV dysfunction and can be significantly improved by CPAP therapy.

## Introduction

Sleep-disordered breathing (SDB) is often reported to cause cardiovascular diseases due to mechanisms, such as intermittent nighttime hypoxemia, followed by oxidative stress, endothelial dysfunction, arousal, sympathetic hyperactivity, changes in intrathoracic pressure, and right and left ventricular (LV) afterload [1–4]. Even patients with SDB who have not developed the cardiovascular disease are likely to have latent and subclinical myocardial dysfunction.

The sleep apnea cardiovascular endpoint (SAVE) study reported negative results regarding the effectiveness of continuous positive airway pressure (CPAP) treatment for obstructive sleep apnea (OSA). However, because of the large number of patients with poor adherence in the target group, the results were carefully interpreted [5]. Numerous studies reported that myocardial dysfunction in SDB (mainly OSA) is based on LV diastolic dysfunction [6–9]. In recent years, LV global longitudinal strain (GLS) measured by the two-dimensional speckle tracking technique has attracted attention as an index of latent and subclinical heart failure [10].

In the past, errors in GLS measurement were problematic because of the echocardiographic equipment and analysis software used. However, recently, the European Association of Cardiovascular Imaging (EACVI)/American Society of Echocardiography (ASE) Industry Task Force published the guidelines for measurement methods. Therefore, errors have virtually disappeared [11–14].

Although the normal values of GLS are still controversial, they have been reported in previous meta-analyses (normal values ranged; −15.9% to −22.1%) [15]. Potter and Marwick classified cardiac dysfunction according to the absolute value of GLS as follows: GLS<8%, very severe; GLS<12%, severe; GLS 12–16%, reduced; GLS 16–18%, borderline; GLS 18–20%, normal; and GLS>20%, supranormal [16].

Only a few reports are available on latent and subclinical heart failure in patients with SDB who do not have cardiovascular disease. Few reports have evaluated whether or not CPAP treatment improves myocardial dysfunction. In our study, latent myocardial dysfunction in patients with SDB was evaluated using GLS (cut-off level >−18%), and changes before and after CPAP treatment were examined.

The purposes of this retrospective cohort study were (1) to clarify the association of SDB and LV function measured with echocardiography and two-dimensional strain analysis in patients without structural heart diseases and (2) to determine the effects of CPAP therapy on measurable LV functional parameters.

## Methods

### Patients and follow-up

**Fig. 1:**
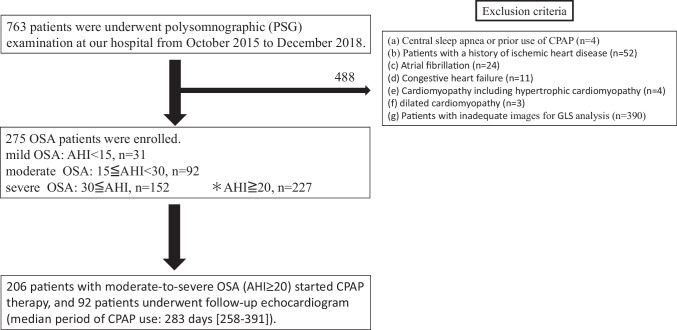
Flow chart of patient selection

In a retrospective study, 275 patients with GLS measured by echocardiography were analyzed after exclusions. These patients were diagnosed with SDB (mainly included OSA) and underwent overnight polysomnographic (PSG) examination at our hospital from October 2015 to December 2018. SDB was clinically diagnosed when overnight PSG examination confirmed apnea-hypopnea index (AHI) ≥5. All study patients underwent baseline echocardiography within 6 months of PSG. The study subjects were patients with OSA and at risk for heart failure who had not yet developed structural heart changes. The exclusion criteria were: (a) central sleep apnea; (b) patients with a history of ischemic heart disease (*n* = 52); atrial fibrillation (*n* = 24), or congestive heart failure (*n* = 11); (c) known cardiomyopathy including hypertrophic cardiomyopathy (*n* = 4) and dilated cardiomyopathy (*n* = 3); (d) patients with inadequate images for GLS analysis (*n* = 390); and (e) prior use of CPAP. Patients were divided into three groups: mild (AHI<15, *n* = 31), moderate (15<AHI<30, *n* = 92), and severe OSA (AHI≥30, *n* = 152), and baseline echocardiographic parameters including GLS were compared. Under the medical insurance system in Japan, CPAP treatment is indicated for OSAS patients with AHI ≥ 20. There were 227 patients with AHI ≥20 in this study. 21 patients were not started on CPAP because they refused treatment. Some patients who refuse CPAP treatment or with 15 ≤ AHI < 20 are started on an oral appliance. A total of 206 patients with moderate to severe OSA (AHI≥20) started CPAP therapy, and 92 patients underwent follow-up echocardiogram (median period of CPAP use: 283 days [258–391]) (Fig. 1). All patients used the same CPAP device (AirSense; ResMed, San Diego, CA 92123 USA). CPAP titration to adjust the optimal pressure was undertaken within a few weeks after its initiation by PSG (of the 92 patients, 87 performed PSG with CPAP therapy). Clinical follow-up examinations were performed after more than six months of CPAP initiation. Patients who were on antihypertensive drugs at the time of baseline echocardiography were defined as hypertensive, and those taking oral hypoglycemic drugs or insulin were defined as diabetic. The study protocol was performed in accordance with the Declaration of Helsinki and was approved by the Ethics Committee of Hamamatsu Red Cross Hospital. Notifications regarding the use of patient information were carried out in the outpatient clinic.

### PSG

PSG is the gold standard for the evaluating and diagnosing sleep disorders [17]. The severity of sleep apnea was classified according to AHI which is the number of apneas (≥90% decrease in airflow) and hypopneas (30% reduction in airflow with ≥3% decrease in O_2_ saturation or on arousal) for a minimum of 10 s. In PSG, 5–14 events/h are defined as mild sleep apnea; 15–29 events/h, moderate; and >30 events/h, severe sleep apnea [18, 19]. Obstructive AHI was calculated from the sum of obstructive events (excluding mixed and central apneas and hypopneas). In this study, patients with obstructive AHI> central AHI were defined as OSA.

### Transthoracic echocardiography

Echocardiogram was performed in almost all patients who underwent PSG examination during the study period. Of the 763 patients who underwent PSG, 390 were excluded because insufficient images were obtained for GLS analysis. Trained echocardiographers performed comprehensive echocardiographic examinations according to the American Society of Echocardiography (ASE) guidelines and using commercially available equipment in all subjects (EPIQ 7; Philips Medical Systems, Andover, MA, USA).

### Two-dimensional speckle tracking analysis

Speckle tracking analysis was performed on two-dimensional images using offline software (Q lab, version 13.0; Philips Medical Systems) by two independent observers blinded to the clinical data. The frame rate was optimized to achieve 60–80 Hz. GLS analysis was performed according to the consensus document by ASE and the European Association of Cardiovascular Imaging (12). Detection of fiducial landmarks (annulus and apex) and tracing of LV endocardial border from three apical views (apical four-, two-, three-chamber views) were automatically performed by using TOMTEC software (Autostrain) to obtain GLS. Two independent observers evaluated the tracking quality and performed adjustments manually if needed. GLS value of −18% or higher was defined as cardiac dysfunction with reference to past literature [15, 16].

### Adherence

The adherence to CPAP treatment was evaluated using remote monitoring software (NemLink; Teijin Pharma, Tokyo, Japan). Nonadherence is defined as a mean of ≤4 h of use per night and/or monthly use <70% on a follow-up echocardiogram [20, 21].

### Statistical analyses

Values were expressed as the mean ± standard deviation if normally distributed or median (interquartile range) if not. Categorical data were compared between the groups using a chi-square test, and one-way analysis of variance was used for continuous values, as appropriate. Paired comparisons before and after CPAP therapy were performed using paired *t*-tests. Statistical significance was set at *p* <0.05. Logistic regression analysis was performed to determine the significant variables associated with predicting cardiac dysfunction (GLS > −18%). Only variables that proved to be significant (*p* <0.10 in the univariate analysis) were considered candidates for the final multivariate model, determined using a forward stepwise variable selection procedure. All statistical analyses were performed using EZR (Saitama Medical Center, Jichi Medical University, Saitama, Japan). Bland–Altman analysis was conducted using Microsoft Excel.

## Results

### Baseline characteristics and echocardiogram parameters

Baseline characteristics did not differ among the three groups (Table 1). Detailed echocardiographic parameters for the three groups are shown in Table 2. These parameters did not differ among groups except for posterior wall dimension. GLS was significantly reduced among the patients with moderate and severe OSA compared with mild OSA (mild: −19.3±2.8%; moderate: −17.8±3.1; severe: −18.0±2.6; *p*=0.038; Table 2). The proportion of patients with GLS ≥ −18% was significantly higher among the patients with moderate to severe OSA than among those with mild OSA (Table 2). Logistic regression analysis identified sex (male) and moderate to severe OSA as significant predictors of cardiac dysfunction (GLS > −18%, Table 3).

**Table 1:** Baseline characteristics of patients with mild, moderate, and severe OSA

Variable	AHI<15 (*n*=31)	15≦AHI<30 (*n*=92)	AHI≧30 (*n*=152)	*p* value
BMI (kg/m^2^)	23.3 ± 2.7	24.9 ± 3.4	†‡25.9 ± 4.2	0.001
Hypertension, *n* (%)	14 (46)	42 (46)	72 (47)	0.953
Diabetes, *n* (%)	0 (0)	9 (10)	20 (13)	0.069
<Medications>				
Aspirin, *n* (%)	1 (3.2)	2 (2.2)	5 (3.3)	0.887
Bate blockers, *n* (%)	1 (3.2)	3 (3.3)	9 (5.9)	0.771
ACEI, *n* (%)	0 (0)	0 (0)	6 (3.9)	0.123
ARB, *n* (%)	10 (32)	34 (37)	50 (33)	0.741
Ca blocker, *n* (%)	11 (36)	31 (34)	57 (38)	0.872
Statins, *n* (%)	3 (9.7)	15 (17)	29 (19)	0.47
<PSG data>				
AHI, events/hour	10.2 ± 2.8	23.6 ± 5.7	47.1 ± 13.8	
Lowest SpO2 (%)	84.7 ± 7.2	†79.2 ± 6.6	†‡74.6 ± 10.1	<0.001
<Laboratory data>:				
Creatinine (mg/dL)	0.82 [0.68–089]	0.79 [0.71–0.90]	0.83 [0.75–0.95]	0.145
eGFR (mL/min/1.73 m^2^)	72.3 ± 13.2	75.3 ± 15.4	71.8 ± 16.4	0.232
HbA1c (%)	5.6 ± 0.4	5.9 ± 0.8	5.9 ± 0.7	0.09
BNP (pg/mL)	20.1 [10.4–43.2]	12.9 [6.8–22.5]	13.5 [5.8–27.0]	0.264

Continuous data are expressed in mean (standard deviation) or median [Q2-Q3], and frequencies are expressed as numbers (percentage)

*OSA* obstructive sleep apnea, *BMI* body mass index, *AHI* apnea hypopnea index, *SpO2* oxygen saturation

Posthoc Tukey’s test and posttest Mann-Whitney *U* test with Bonferroni’s correction: †*p*<0.05 compared with mild OSA, ‡*p*<0.05 compared with moderate OSA

**Table 2:** Baseline conventional, tissue Doppler, and global longitudinal strain values of patients in three groups

	AHI < 15 (*n*=31)	15≦AHI<30 (*n*=92)	AHI≧30 (*n*=152)	*p* value
LVDd (mm)	46.8 ± 4.3	47.6 ± 4.9	47.3 ± 4.1	0.671
LVDs (mm)	28.3 ± 3.7	29.1 ± 4.8	28.8 ± 3.9	0.616
IVSd (mm)	8.9 ± 1.0	8.9 ± 1.2	8.9 ± 1.2	0.949
PWd (mm)	9.1 ± 1.2	9.1 ± 1.0	‡9.4 ± 1.0	0.035
LVEF (%)	69.9 ± 5.7	69.2 ± 6.3	69.3 ± 6.4	0.834
Mitral *E*/*A*	1.0 ± 0.3	0.9 ± 0.3	0.9 ± 0.3	0.805
Mitral DT (ms)	248 ± 63	236 ± 51	248 ± 64	0.274
*e*^′^ (m/sec)	0.072 ± 0.022	0.067 ± 0.017	0.067 ± 0.018	0.277
*E*/*e*^′^	11.4 ± 3.1	11.2 ± 3.1	11.4 ± 4.9	0.95
LAD (mm)	32.8 ± 3.8	33.2 ± 4.3	34.1 ± 3.7	0.126
LAVI (mL)	18.2 ± 5.4	18.2 ± 5.9	19.6 ± 7.4	0.321
GLS (%)	−19.3 ± 2.8	†−17.8 ± 3.1	−18.0 ± 2.6	0.038
GLS >−18%, *n* (%)	8 (25)	†48 (52)	74 (49)	0.033
GLS >−16%, *n* (%)	2 (7)	†27 (29)	37 (24)	0.026

Data are expressed in mean (standard deviation)

*LVDd* left ventricular diastolic diameter, *LVDs* left ventricular end-systolic diameter, *IVSd* interventricular septum dimension, *PWd* posterior wall dimension, *LVEF* Left ventricular ejection fraction, *E*/*A* the ratio between early and late diastolic mitral flow velocities, *e*^′^ tissue Doppler diastolic annular velocity, *E*/*e*^′^ the ratio between early diastolic mitral inflow velocity and early diastolic annular velocity, *LAD* left atrial diameter, *LAVI* left atrial volume index, *GLS* global longitudinal strain

Posthoc Tukey’s test and posttest Mann-Whitney *U* test with Bonferroni’s correction: †*p*<0.05 compared with mild OSA, ‡*p*<0.05 compared with moderate OSA

**Table 3:** Logistic regression analysis for the predictors of cardiac dysfunction (GLS > −18%)

	Univariate		Multivariate	
	OR (95% CI)	*p* value	OR (95% CI)	*p* value
Age per 1 year	1.01 (0.99–1.03)	0.315		
Sex (male)	2.13 (1.14–3.97)	0.017	2.01 (1.07–3.78)	0.03
BMI per 1.0	1.00 (0.94–1.06)	0.998		
Hypertension	1.03 (0.64–1.65)	0.905		
Diabetes	1.05 (0.48–2.26)	0.909		
Moderate-to-severe OSA	3.02 (1.31–7.00)	0.01	2.83 (1.22–6.61)	0.016

*CI* confidence interval, *OR* odds ratio. Other abbreviations as in Tables 1 and 2

Moderate-to-severe OSA was an independent predictor of cardiac dysfunction

### CPAP effects on LV function

CPAP usage and adherence data are presented in Table 4. The effects of CPAP on the LV structural and functional parameters are shown in Table 5. GLS significantly improved after CPAP therapy in patients with moderate to severe OSA (GLS: −18.1±2.7% to −19.0±2.8%, *p*=0.004). In addition, significant improvement in GLS was confirmed in patients with good adherence to CPAP therapy (adherence group: −18.1±2.7% to −18.9±3.0%, *p*=0.011; nonadherence group: −18.5±2.5% to −19.3±2.5%, *p*=0.175) (Table 6). The proportion of patients with GLS −16% or higher was significantly reduced by CPAP treatment but not associated with tolerability of CPAP treatment (Table 6).

**Table 4:** CPAP usage and adherence date

All patients (*n* = 92)	
CPAP usage period (days)	283 [258–391]
CPAP usage rate (%)	96.6 [83.6–100]
CPAP use 4 h/d for 70% of the nights per month (%)	83.3 [60.0–96.6]
CPAP-PSG (*n*=88)	
AHI	2.8 ± 4.0
Lowest SpO2 (%)	90.5 ± 5.0

The adherence to CPAP treatment was evaluated using remote monitoring software on a follow-up echocardiogram. CPAP titration to adjust the optimal pressure was undertaken at least within a few weeks after its initiation by PSG (of the 92 patients, 87 performed PSG with CPAP therapy)

**Table 5:** Patient’s echocardiographic data before and after treatment with CPAP

	Baseline	Follow-up	*p* value
LVEF (%)	70.0 ± 5.3	69.0 ± 4.6	0.084
Mitral *E*/*A*	1.0 ± 0.4	1.0 ± 0.3	0.405
*e*^′^ (m/sec)	0.068 ± 0.018	0.069 ± 0.018	0.267
*E*/*e*^′^	11.2 ± 3.3	11.3 ± 2.8	0.929
LAVI (mL)	19.9 ± 6.0	20.9 ± 8.8	0.288
GLS (%)	−18.1 ± 2.7	−19.0 ± 2.8	0.004
GLS > −18%, *n* (%)	47 (51)	33 (36)	0.053
GLS > −16%, *n* (%)	22 (24)	10 (11)	0.031

Data are expressed in mean (standard deviation)

*LVEF* left ventricular ejection fraction, *E/A* the ratio between early and late diastolic mitral flow velocities, *e*^′^ tissue Doppler diastolic annular velocity, *E*/*e*^′^ the ratio between early diastolic mitral inflow velocity and early diastolic annular velocity, *LAVI* left atrial volume index, *GLS* global longitudinal strain

**Table 6:** Comparison of global longitudinal strain (GLS) improvement after continuous positive airway pressure (CPAP) treatment between adherence and nonadherence groups

	Baseline	Follow-up	*p* value
Adherence, *n*=65			
GLS (%)	−18.1 ± 2.7	−18.9 ± 3.0	0.011
GLS>−18%, *n* (%)	35 (54)	26 (40)	0.16
GLS>−16%, *n* (%)	16 (25)	8 (12)	0.112
Nonadherence, *n*=27			
GLS (%)	−18.5 ± 2.5	−19.3 ± 2.5	0.175
GLS>−18%, *n* (%)	12 (44)	7 (26)	0.254
GLS>−16%, *n* (%)	6 (22)	2 (7)	0.25

Significant improvement in GLS was confirmed, particularly in patients with good adherence to CPAP therapy. Nonadherence is defined as a mean of ≤4 hours of use per night and/or monthly use <70%

### Reproducibility and reliability of GLS

**Fig. 2:**
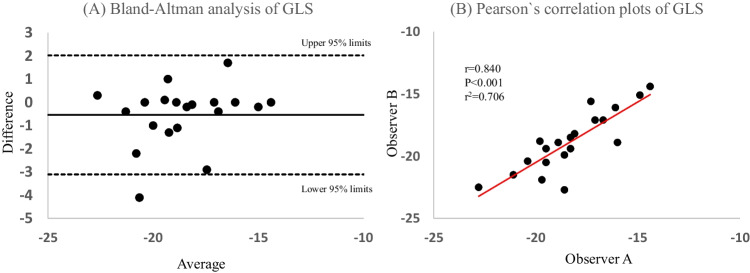
Bland-Altman and Pearson’s correlation plots. **A** Bland-Altman plots of global longitudinal strain (GLS); **B** Pearson’s correlation of GLS measurements obtained from two independent observers in 20 patients. The horizontal solid line shows the mean of the differences (=bias) between the two observers, and the dotted horizontal lines show the upper and lower 95% limits of agreement (= bias ± 1.96 × SD). *r*: correlation coefficient; *r*^2^: squared *r* for the goodness of fit

Pearson’s correlation and Bland-Altman analysis were performed for intraobserver variability of GLS from 20 randomly selected patients (Fig. 2).

## Discussion

This study investigated latent and subclinical myocardial dysfunction in patients with OSA without cardiac disease and the efficacy of CPAP treatment for damage. The results showed that GLS was lower in patients with moderate to severe OSA than in those with mild OSA and was improved by CPAP treatment. In addition, GLS improved significantly in patients who tolerated CPAP therapy.

LV ejection fraction (LVEF) is the most commonly used cardiac function index, and reduced LVEF worsens cardiovascular prognosis. However, previous reports have shown that LVEF >45–50% is not correlated with cardiovascular mortality [22]. LVEF does not directly reflect the myocardial contractility but is an index calculated from changes in the area of the LV lumen. GLS is a highly reliable and reproducible index for evaluating LV systolic function and is a strong independent indicator of cardiac prognosis [23, 24]. GLS can detect myocardial injury with higher sensitivity than LVEF, and its use is recommended in European and American cardiovascular-related guidelines as an index with excellent reproducibility [25, 26]. GLS is more appropriate than LVEF as an index for detecting myocardial damage before the appearance of structural abnormalities in the heart, such as the subject of this study.

Previous studies showed that GLS is reduced in patients with very severe OSA compared with matched controls [27]. According to Altekin et al., GLS was significantly lower in patients with OSA than in healthy subjects and decreased along with the OSA severity [28]. In these reports, the number of subjects was small, and the GLS value, which is a criterion for cardiac dysfunction, was not defined.

In this study, 275 patients, the largest number of patients with OSA evaluated for GLS, were included and a GLS value of −18% or higher was considered indicative of cardiac dysfunction. Compared to the results of previous studies, the difference in GLS values among the three groups is small, and it is controversial whether or not the difference is clinically significant. GLS is useful as an index for detecting early-stage myocardial damage, and in recent years, it has also become an important index for follow-up of Cancer Therapeutics-Related Cardiac Dysfunction. According to the European Society of Cardiology (ESC), American Society of Echocardiography (ASE) and European Association of Cardiovascular Imaging (EACVI) guidelines, 15% or more relative percentage reduction of GLS from baseline may suggest risk of cardiotoxicity [26, 29]. Based on these reports, the difference in GLS between the three groups confirmed in this study suggests that moderate to severe OSA can cause subclinical myocardial damage. Multivariate analysis showed that moderate to severe OSA was an independent factor in lowering GLS with or without hypertension. This suggested that the underlying cardiac dysfunction is caused by a mechanism unique to the pathological condition of OSA, such as intermittent hypoxemia and changes in intrathoracic pressure during the night.

The efficacy of CPAP treatment for subclinical and latent LV dysfunction has been reported by several studies [30–32]. In these studies, patient adherence to CPAP treatment was not evaluated; therefore, whether Cor not PAP treatment was effective is debatable.

The SAVE study, reported in 2016, was a randomized controlled trial that examined the effectiveness of CPAP therapy in the secondary prevention of cardiovascular events in patients with moderate to severe OSA and cardiovascular and cerebrovascular diseases [5]. There were no differences in cardiovascular events between the CPAP and control groups, and the effect of CPAP on cardiovascular events was not proven. Although subanalysis was performed by dividing the CPAP use time into 4 h or more and less than 4 h, the rate of cardiovascular events was lower in the group administered for 4 h or more than in the usual care group. In the present study, adherence to CPAP treatment was evaluated using remote monitoring software (NemLink; Teijin Pharma, Tokyo, Japan).

Among patients with good adherence, GLS improved significantly with CPAP treatment. However, the improvement in the proportion of patients with GLS −18% or higher was not associated with the tolerability of CPAP treatment. It may not have been possible to normalize cardiac function within the observation period of this study, as it may take more time for CPAP treatment to improve cardiac function in patients with impaired cardiac function.

Patients with OSA have potentially advanced cardiac dysfunction, which is considered a significant risk factor for future heart disease. In stage A patients with chronic heart failure, evaluating OSA as a risk factor for heart failure and performing therapeutic interventions is essential.

This study has three main limitations. First, it was a retrospective observational study, and many patients were excluded because of insufficient echocardiographic images to analyze GLS (*n*=390). In addition, more than half of the patients who started CPAP had not undergone follow-up echocardiography or had poor images. Second, patients who took antihypertensive drugs at the first visit were defined as hypertensive patients in this study. Therefore, subclinical hypertensive patients may have been included in the group of patients without hypertension. Third, the CPAP use status during the one month before follow-up echocardiography was used as the criterion for adherence. Adherence had not been evaluated in any period since the start of CPAP therapy.

## Conclusion

Moderate to severe OSA is associated with LV dysfunction which is significantly improved by CPAP therapy.

Cardiologists would be well advised to understand the cardiovascular risk of obstructive sleep apnea and the potential to improve a patient’s condition through CPAP treatment in routine clinical practice.

## Data Availability

The datasets generated during and/or analyzed during the current study are available from the corresponding author on reasonable request.
